# Atypical Lyme Neuroborreliosis Presenting With Ventriculitis and Cerebritis: A Case Report

**DOI:** 10.7759/cureus.115883

**Published:** 2026-09-07

**Authors:** Ana Albuquerque, Ana Claudia Cunha, Odete Gomes, Luisa Pinto, Márcia Pinto

**Affiliations:** 1 Intensive Care Unit, Centro Hospitalar de Leiria, Leiria, PRT; 2 Intensive Care Unit, Unidade Local de Saúde da Região de Leiria, Leiria, PRT

**Keywords:** central nervous system infection, cerebrospinal fluid, lyme disease, meningoencephalitis, neuroimaging

## Abstract

Lyme neuroborreliosis is a neurological manifestation of infection by *Borrelia burgdorferi* sensu lato and most commonly presents with lymphocytic meningitis, cranial neuropathy, or painful radiculitis. Central nervous system parenchymal involvement is uncommon, and ventriculitis is rarely reported. We describe a man in his late 70s who presented with acute confusion, dysarthria, and left-sided weakness following several days of severe lower-limb pain and progressive mobility impairment. His neurological status rapidly deteriorated, with secondary seizures requiring invasive mechanical ventilation and intensive care support. Laboratory evaluation demonstrated a marked systemic inflammatory response, thrombocytopenia, acute kidney injury, and cholestatic liver dysfunction. Cerebrospinal fluid (CSF) analysis revealed pleocytosis with initial neutrophilic predominance, markedly elevated protein, and preserved glucose. Routine bacterial cultures and molecular testing for common central nervous system pathogens were negative. Anti-*Borrelia* IgG and IgM antibodies were detected in CSF, although a paired CSF-serum antibody index was unavailable. Brain magnetic resonance imaging demonstrated inflammatory material within the occipital horns of the lateral ventricles, bilateral leptomeningeal involvement, and focal parenchymal lesions with restricted diffusion suggestive of cerebritis. In the appropriate clinical context, these findings supported a diagnosis of probable Lyme neuroborreliosis with an unusual ventriculitis-like presentation. Despite prolonged antimicrobial therapy and partial improvement in CSF and radiological inflammatory findings, neurological recovery remained limited, and the patient subsequently died following ventilator-associated pneumonia complicated by septic shock. This case illustrates an uncommon and severe central nervous system presentation potentially associated with Lyme neuroborreliosis and highlights both the diagnostic value and limitations of CSF serology when demonstration of intrathecal antibody synthesis is unavailable.

## Introduction

Lyme borreliosis is a tick-borne multisystem infection caused by spirochetes belonging to the *Borrelia burgdorferi *sensu lato complex and is the most common tick-borne infectious disease in Europe [1,2]. Neurological involvement occurs in approximately 3%-15% of infections and encompasses a broad spectrum of peripheral and central nervous system (CNS) manifestations [1,2].

In European adults, painful meningoradiculitis, cranial neuropathies, and lymphocytic meningitis are characteristic manifestations of Lyme neuroborreliosis [1]. Direct CNS involvement is considerably less common. In a systematic literature review and Scandinavian cohort, encephalitis represented an uncommon manifestation of Lyme neuroborreliosis and was associated with a heterogeneous clinical presentation that included cognitive impairment, focal neurological deficits, and seizures [3].

Neuroimaging findings are similarly heterogeneous and are often normal or nonspecific [2,4,5]. Reported abnormalities include meningeal and cranial nerve enhancement, inflammatory parenchymal lesions, myelitis, vasculitic changes, and abnormalities involving the white matter [4,5]. In patients presenting with inflammatory meningoencephalitic, ventriculitis-like, or cerebritis-like abnormalities, the differential diagnosis includes other bacterial or viral CNS infections, autoimmune or inflammatory encephalitis, and vascular disorders, emphasizing the need to integrate neuroimaging with clinical, cerebrospinal fluid (CSF), and microbiological findings [4,5].

The diagnosis of European Lyme neuroborreliosis relies on the combination of a compatible neurological syndrome, CSF inflammation, and evidence of intrathecal production of *Borrelia*-specific antibodies. According to the European Federation of Neurological Societies (EFNS) criteria, definite Lyme neuroborreliosis requires fulfillment of all three criteria, whereas fulfillment of two criteria supports a diagnosis of possible Lyme neuroborreliosis [6]. We describe a severe neurological syndrome fulfilling criteria for possible Lyme neuroborreliosis and associated with an unusual MRI pattern characterized by intraventricular inflammatory material, leptomeningeal involvement, and focal lesions suggestive of cerebritis.

## Case presentation

A man in his late 70s with a medical history of arterial hypertension was brought to the Emergency Department by prehospital emergency services because of acute confusion, dysarthria, and left-sided weakness. The focal neurological symptoms had been recognized approximately two hours before presentation, prompting activation of the acute stroke pathway.

The patient had no functional limitations and was independent in activities of daily living before symptom onset. During the preceding week, he had experienced an unexplained fall followed by severe neuropathic-type pain in the lower limbs. He had been evaluated in primary care and subsequently in an urgent care setting because of persistent pain. During the following days, progressive impairment in mobility and left-sided weakness developed. No history of tick bite or erythema migrans was documented.

On arrival, blood pressure was 151/80 mmHg, heart rate was 105 beats/minute, peripheral oxygen saturation was 99% on room air, and blood glucose was 140 mg/dL. Neurological examination demonstrated dysarthria, left-sided hemiparesis, and impaired consciousness, with a Glasgow Coma Scale score of 10 (E3V2M5). Marked discomfort and pain were elicited by mobilization of the lower limbs. The abdomen was mildly distended and tender, particularly in the right upper quadrant.

Initial laboratory testing demonstrated a marked inflammatory response associated with hematological, renal, and hepatic dysfunction. The principal abnormalities included leukocytosis, thrombocytopenia, markedly increased C-reactive protein and procalcitonin concentrations, severe acute kidney injury, and predominantly conjugated hyperbilirubinemia with an elevated alkaline phosphatase level (Table 1).

**Table 1:** Initial laboratory findings.

Laboratory parameter	Patient value	Reference range
White blood cell count (×10^9^/L)	24.8	4.0-10.0
Neutrophils (×10^9^/L)	17.57	2.0-7.0
Platelets (×10^9^/L)	48	150-400
C-reactive protein (mg/L)	257	0-5
Procalcitonin (ng/mL)	40	<0.5
Creatinine (mg/dL)	4.79	0.67-1.18
Urea (mmol/L)	47.5	17-43
Alkaline phosphatase (U/L)	313	30-120
Total bilirubin (µmol/L)	83.5	5-21
Direct bilirubin (µmol/L)	56.3	<3.5
Indirect bilirubin (µmol/L)	27.2	1.7-17.1

Urinalysis demonstrated leukocyte esterase corresponding to approximately 500 leukocytes/µL, proteinuria, hematuria, bilirubinuria, and increased urobilinogen, with negative nitrites. Urinary investigation for *Leptospira* was negative.

Initial non-contrast brain computed tomography (CT) demonstrated a right nucleocapsular hypodensity of uncertain age, without acute intracranial hemorrhage, significant mass effect, or cerebral herniation (Figure 1). The findings did not adequately explain the rapidly progressive neurological syndrome.

**Figure 1:**
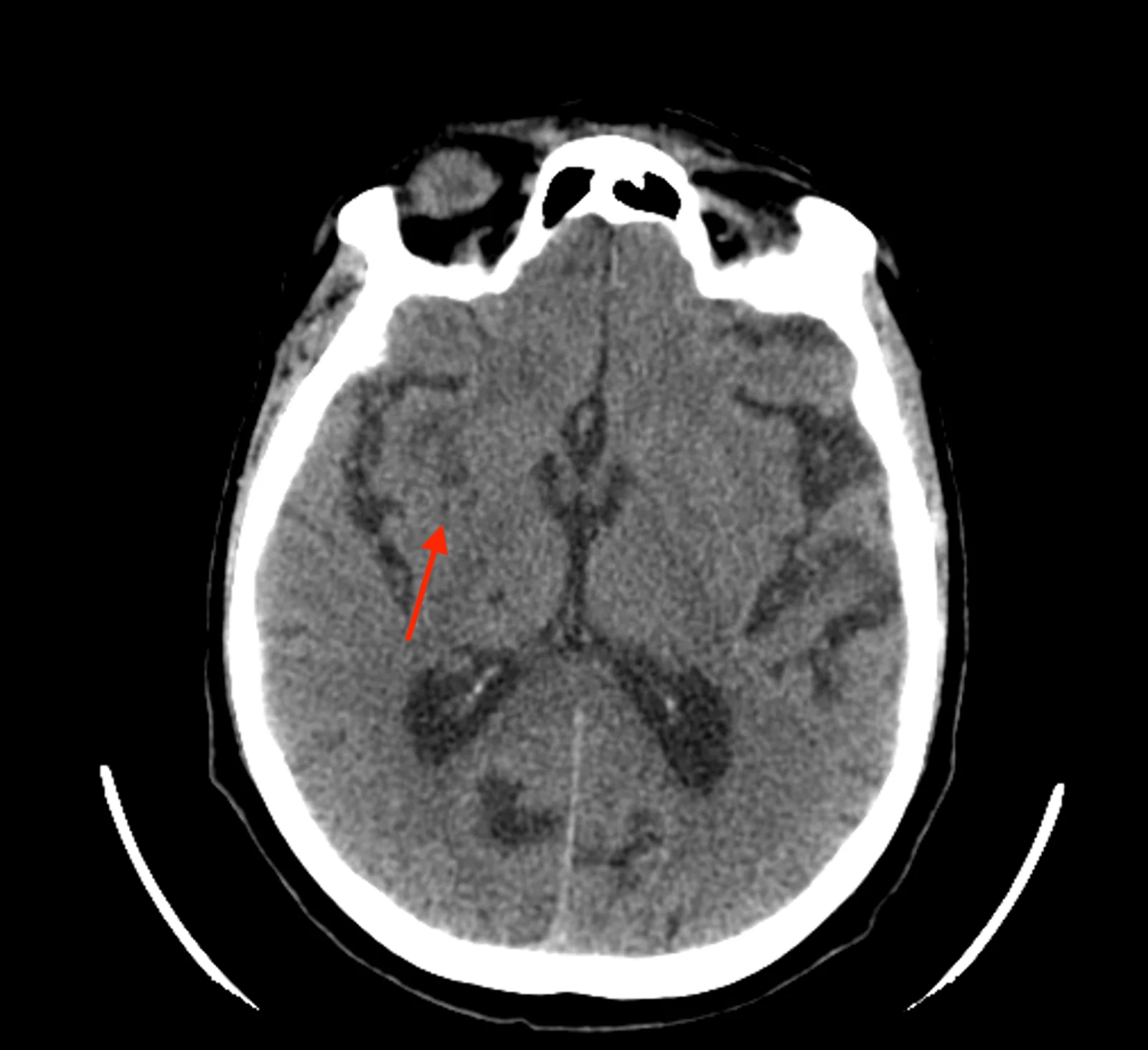
Initial non-contrast brain computed tomography showing a right nucleocapsular hypodensity of uncertain age (red arrow).

Given the severe neuropathic-type lower-limb pain preceding the acute neurological deterioration, an inflammatory radicular process was also considered. Neurological status subsequently deteriorated, and secondary seizures occurred, requiring endotracheal intubation and invasive mechanical ventilation.

Because of severe thrombocytopenia, platelet transfusion was administered before lumbar puncture. CSF was obtained under increased opening pressure (26 cmH_2_O) and had a yellowish appearance (Figure 2).

**Figure 2:**
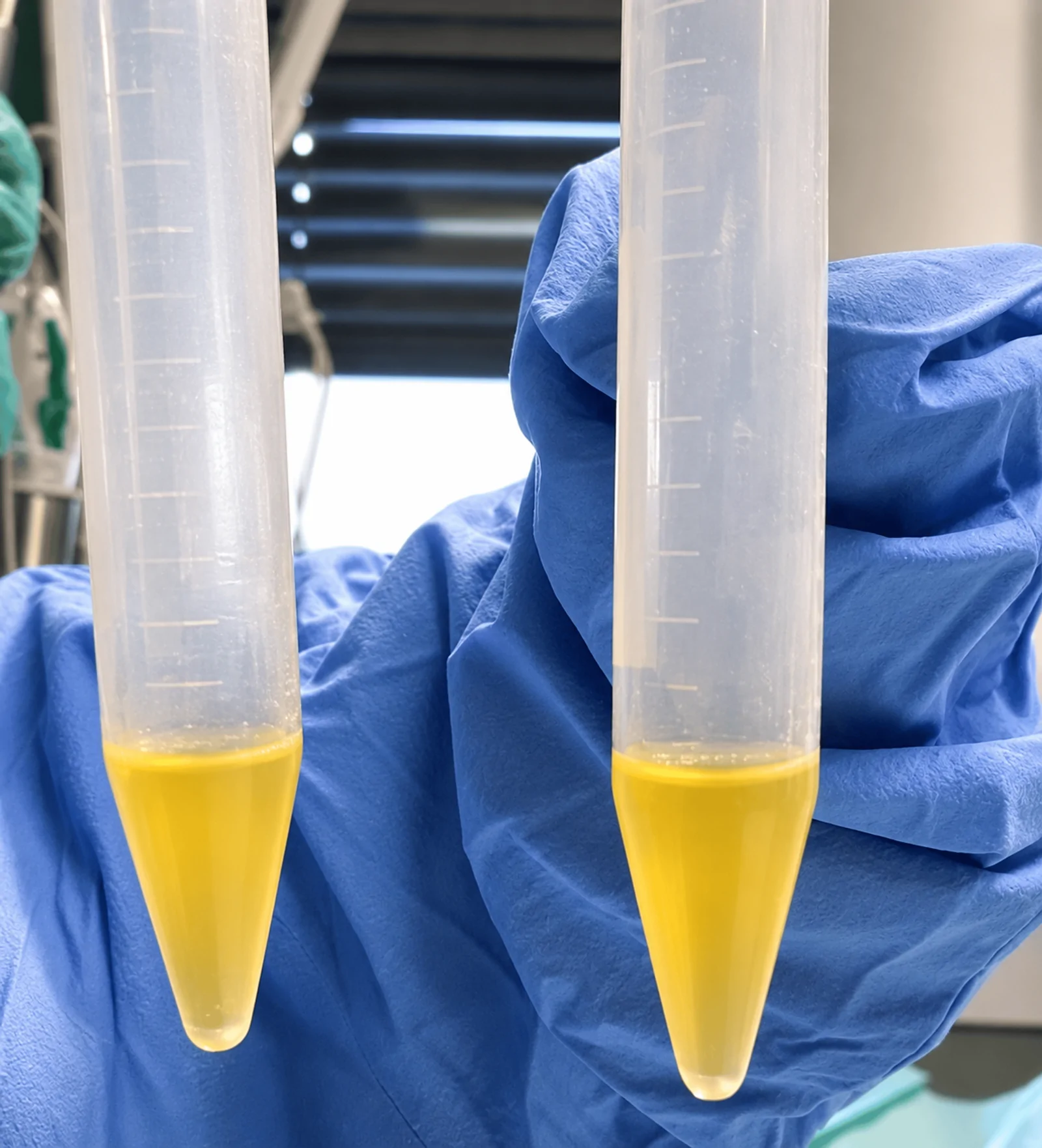
Yellowish appearance of the cerebrospinal fluid obtained during the initial lumbar puncture.

The initial CSF showed 190 leukocytes/mm^3^, with 76% polymorphonuclear (PMN) cells and 24% mononuclear cells, 320 erythrocytes/mm^3^, glucose 69 mg/dL, and a markedly elevated protein level of 604 mg/dL. Gram stain showed no bacteria, and aerobic culture was negative (Table 2).

**Table 2:** Serial cerebrospinal fluid (CSF) findings.

Parameter	Initial CSF	Day 21	Subsequent CSF	Reference range
Appearance	Yellowish	Clear	Clear	Clear
Leukocytes (cells/mm^3^)	190	28	2	0-5
Polymorphonuclear cells (%)	76	33	0	<5
Mononuclear cells (%)	24	67	100	<5
Erythrocytes (cells/mm^3^)	320	3	45	0
Glucose (mg/dL)	69	110	181	40-70
Total protein (mg/dL)	604	179	188	15-45
Gram stain	No bacteria observed	No bacteria observed	No bacteria observed	Negative
Aerobic culture	Negative	Negative	Negative	Negative

Serial lumbar punctures demonstrated a marked reduction in leukocyte count and a shift from PMN to mononuclear predominance, although CSF protein remained elevated. The final clinical record confirms an evolution from 190 cells/μL with 76% PMN cells to 28 cells/μL with 67% mononuclear cells, followed by two cells/μL with no PMN cells.

Microbiological and molecular investigations for alternative CNS infections were unrevealing. Bacterial antigen testing for *Escherichia coli* K1, *Haemophilus influenzae* type B, *Neisseria meningitidis*, *Streptococcus agalactiae*, and *Streptococcus pneumoniae* was negative. PCR testing for herpes simplex virus types 1 and 2, varicella-zoster virus, and *Borrelia* was also negative.

CSF immunological testing detected anti-*Borrelia* IgG at 26.0 U/mL, above the laboratory positivity threshold of 11 U/mL, and anti-*Borrelia* IgM at 6.5 U/mL (Table 3).

**Table 3:** Serological and Borrelia-specific findings in CSF and serum, with their respective interpretation. CSF: cerebrospinal fluid; IgG: immunoglobulin G; IgM: immunoglobulin M; PCR: polymerase chain reaction; HIV: human immunodeficiency virus

Test	Result	Interpretation
Cerebrospinal fluid		
*Borrelia* IgG, initial	26.0 U/mL	Positive (>11 U/mL)
*Borrelia* IgM, initial	6.5 U/mL	Positive (>1 U/mL)
*Borrelia* PCR	Negative	Negative
*Borrelia* IgG, follow-up	22.0 U/mL	Positive (>11 U/mL)
*Borrelia* IgM, follow-up	2.7 U/mL	Positive (>1 U/mL)
*Borrelia* antibody index	Not available	Intrathecal antibody synthesis could not be formally demonstrated
Serum		
*Borrelia* IgG	9 U/mL	Negative (<11 U/mL)
*Borrelia* IgM	11.3 U/mL	Positive (>1 U/mL)
*Rickettsia conorii* IgG	Negative	Negative
*R. conorii* IgM	1:80	Positive
*Leptospira* IgM	Negative	Negative
*Coxiella burnetii* phase I IgG/IgM	Negative	Negative
*C. burnetii* phase II IgG/IgM	Negative	Negative
Rose Bengal test (*Brucella abortus*)	Negative	Negative
*Chlamydophila pneumoniae* IgG	23.7 UNT	Positive (>11 UNT)
*C. pneumoniae* IgM	4.1 UNT	Negative (< 11 UNT)
*Mycoplasma pneumoniae* IgG	16.6 U/mL	Negative (<20 U/mL)
*M. pneumoniae* IgM	1.9 U/mL	Negative (<13 U/mL)
*Listeria monocytogenes* IgM	Non-reactive	Negative
Syphilis screening (IgG/IgM)	<0.1 index	Negative

The presence of anti-*Borrelia* antibodies in CSF, in association with compatible neurological manifestations and CSF inflammation, supported Lyme neuroborreliosis. However, because paired CSF-serum testing permitting calculation of a *Borrelia*-specific antibody index was unavailable, intrathecal antibody synthesis could not be formally demonstrated. According to EFNS criteria, which define definite Lyme neuroborreliosis by the presence of compatible neurological symptoms, CSF pleocytosis, and intrathecal production of *Borrelia*-specific antibodies, while two of these three criteria support possible Lyme neuroborreliosis, the present case was therefore classified as possible rather than definite Lyme neuroborreliosis [6].

Blood cultures remained negative, and echocardiography showed no vegetations or other findings suggestive of an endovascular source of septic emboli.

Brain MRI subsequently demonstrated intraventricular material within the occipital horns of the lateral ventricles (Figure 3). Bilateral sulcal and corticopial hyperintensity extending to the perimesencephalic region was suggestive of leptomeningeal inflammatory involvement.

**Figure 3:**
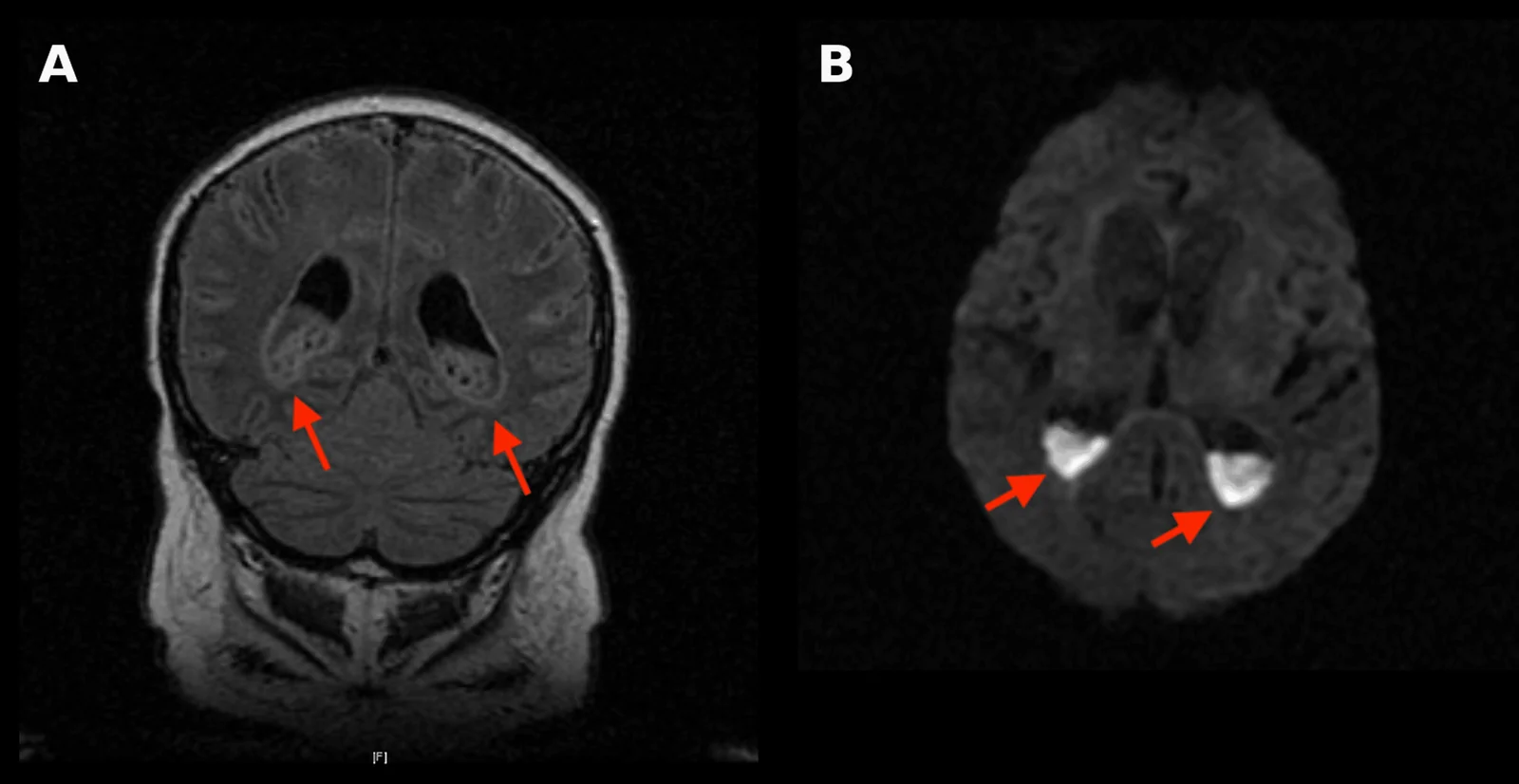
Brain magnetic resonance imaging showing intraventricular inflammatory material. (A) Coronal T2-FLAIR image showing inflammatory material within the occipital horns of the lateral ventricles (red arrows). (B) Axial diffusion-weighted image showing restricted diffusion of the intraventricular material (red arrows), producing a ventriculitis-like imaging appearance. FLAIR: fluid-attenuated inversion recovery

Additional right frontal and left parietal lesions were hyperintense on T2-weighted and fluid-attenuated inversion recovery (FLAIR) sequences and demonstrated restricted diffusion, raising suspicion for focal areas of cerebritis (Figure 4). Additional periventricular, lenticulocapsular, and corona radiata white-matter abnormalities were considered most consistent with chronic microangiopathic disease.

**Figure 4:**
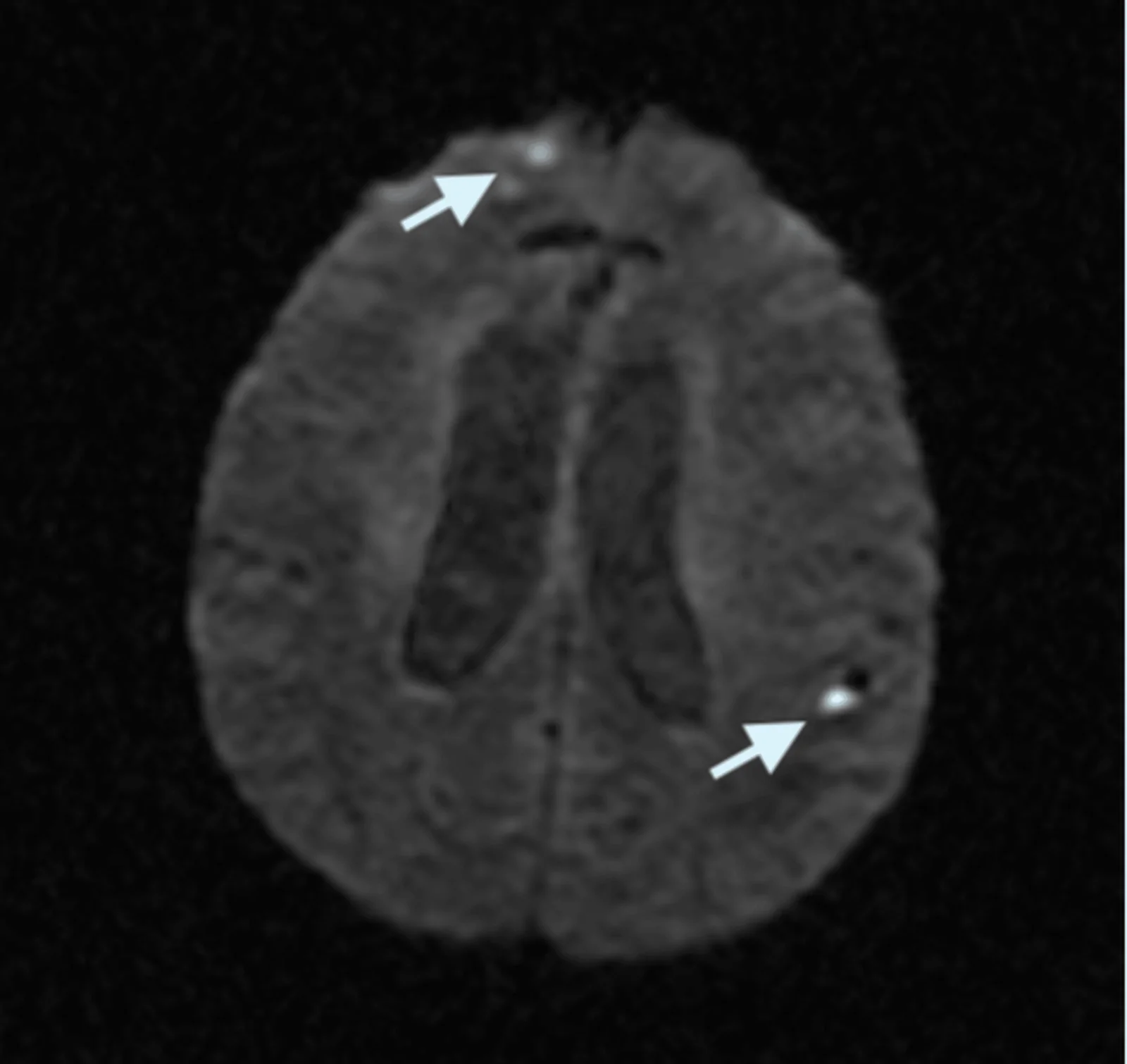
Brain magnetic resonance imaging (MRI), axial diffusion-weighted imaging (DWI), showing focal parenchymal lesions in the right frontal and left parietal lobes with diffusion restriction, suggestive of cerebritis (blue arrows).

Empirical antimicrobial therapy initially included intravenous ampicillin, ceftriaxone, vancomycin, doxycycline, and acyclovir, providing broad coverage for bacterial and viral meningoencephalitis while the diagnostic investigation was ongoing. Acyclovir was discontinued after seven days. Ampicillin and vancomycin were discontinued after 16 days. Doxycycline was continued for 28 days, while ceftriaxone was maintained for a total of six weeks following multidisciplinary discussion because of the severity and persistence of the CNS inflammatory findings. The final clinical record confirms the decision to maintain ceftriaxone for six weeks and the completion of 28 days of doxycycline.

The possibility of neurosurgical intervention and intrathecal antimicrobial treatment was discussed with the respective specialist teams. Neither was considered indicated because, although ventricular enlargement was present, there was no evidence of acute obstructive hydrocephalus requiring CSF diversion, cerebral abscess, or another clear neurosurgical target.

Follow-up MRI initially showed some reduction in the ventricular and sulcal inflammatory abnormalities, without convincing intraparenchymal enhancement suggestive of an overt abscess. Mild posterior periventricular enhancement remained, potentially reflecting ependymal inflammation. Mild ventricular enlargement persisted without clear progression at that stage.

Electroencephalographic monitoring initially documented seizures, prompting anticonvulsant treatment. Subsequent recordings no longer demonstrated seizures under treatment. Despite antimicrobial treatment and partial improvement in CSF and neuroimaging abnormalities, neurological recovery remained limited, and prolonged mechanical ventilation was required. A later MRI showed mild progression of the previously described sulcal and ventricular inflammatory exudate, with slightly increased supratentorial ventricular ectasia.

During the subsequent ICU course, the patient developed ventilator-associated pneumonia complicated by septic shock. Despite antimicrobial therapy and full intensive care support, he deteriorated and died during hospitalization.

## Discussion

Lyme neuroborreliosis encompasses a broad spectrum of neurological manifestations involving both the peripheral and CNS. Painful radiculitis, lymphocytic meningitis, and cranial neuropathy are among its most characteristic European presentations [1,2]. Encephalitic involvement is substantially less common. In a systematic review combined with a Scandinavian cohort, encephalitis represented an uncommon manifestation of Lyme neuroborreliosis and was associated with confusion, focal neurological deficits, aphasia, seizures, and other manifestations of cerebral dysfunction [3].

Several aspects of the present case are noteworthy. First, severe lower-limb pain preceded the acute encephalopathic presentation by several days. Painful radiculitis is a characteristic manifestation of European Lyme neuroborreliosis and forms part of the classical Bannwarth syndrome [1]. Although the severe lower-limb pain preceding the CNS presentation may have been compatible with radicular involvement, the available clinical data do not allow this manifestation to be definitively characterized as Bannwarth syndrome.

Second, the initial CSF profile was unusual for established Lyme neuroborreliosis. Typical CSF findings include pleocytosis, usually with lymphocytic predominance, increased total protein or albumin quotient, and generally preserved glucose [1,6,7]. In a series of adults with acute Lyme neuroborreliosis, lymphocytic pleocytosis was a consistent finding [6]. Our patient instead demonstrated marked neutrophilic predominance at presentation, followed by a progressive transition toward mononuclear predominance on subsequent lumbar punctures. This evolution should therefore be interpreted in the context of an acute inflammatory CNS process rather than as a classical CSF profile of Lyme neuroborreliosis.

Third, the neuroradiological presentation was striking. Neuroimaging in Lyme neuroborreliosis has no single pathognomonic appearance, and its main role is often the identification of complications and exclusion of alternative diagnoses [2,4,5]. Meningeal or cranial nerve enhancement, myelitis, inflammatory parenchymal abnormalities, ischemic lesions associated with vasculitis, and other less common abnormalities have been described [2,4,5].

In the present patient, MRI demonstrated inflammatory material within the occipital horns of the lateral ventricles, associated bilateral sulcal/corticopial signal abnormalities, and focal right frontal and left parietal lesions with diffusion restriction, reported radiologically as possibly related to cerebritis. This combination produced a ventriculitis-like imaging pattern. Although encephalitic and parenchymal manifestations of Lyme neuroborreliosis are well documented, they are uncommon [3,8,9]. We were unable to identify a well-established characteristic pattern of *Borrelia*-associated ventriculitis in the available literature. Therefore, the radiological findings are more appropriately described as an exceptionally unusual ventriculitis-like and cerebritis-like presentation occurring in association with possible Lyme neuroborreliosis, rather than as a recognized characteristic manifestation directly attributable to *Borrelia* infection.

The etiological interpretation requires particular caution. European diagnostic criteria for definite Lyme neuroborreliosis require neurological manifestations compatible with the disease, CSF pleocytosis, and evidence of intrathecal production of *Borrelia*-specific antibodies [1,6]. The present patient fulfilled the first two criteria. Anti-*Borrelia* IgG and IgM were detected in CSF, and serum IgM reactivity was also documented. However, a paired CSF-serum antibody index was unavailable. Consequently, specific intrathecal antibody production could not be formally established. Detection of *Borrelia*-specific antibodies in CSF alone does not necessarily demonstrate intrathecal synthesis because antibodies may enter the CSF from serum, particularly in the presence of blood-CSF barrier dysfunction [1,8].

Accordingly, under the EFNS case definition, the patient fulfilled two of the three diagnostic criteria and was therefore classified as having possible Lyme neuroborreliosis rather than definite Lyme neuroborreliosis [6]. The unusual neuroimaging abnormalities should consequently be interpreted as being associated with possible Lyme neuroborreliosis rather than as definitively attributable to *Borrelia* infection.

A negative *Borrelia* PCR result in CSF does not independently exclude the diagnosis. Direct molecular detection has limited sensitivity in Lyme neuroborreliosis, particularly beyond the earliest phase of neurological infection, and European recommendations emphasize the combined interpretation of the clinical syndrome, CSF inflammation, and intrathecal antibody production [1,6,8].

Alternative infectious diagnoses were also considered. The isolated *Rickettsia conorii* IgM positivity, in the absence of IgG reactivity and of a compatible clinical syndrome, was considered insufficient to support a diagnosis of acute rickettsial infection. Similarly, isolated *Chlamydophila pneumoniae* IgG positivity with negative IgM was interpreted as evidence of previous exposure rather than acute infection. These findings were therefore considered unlikely to explain the neurological presentation. The remaining extensive microbiological investigation did not identify an alternative bacterial or viral CNS pathogen.

The antimicrobial regimen reflected the severity of illness and the broad initial differential diagnosis. Ampicillin, ceftriaxone, vancomycin, doxycycline, and acyclovir were initially prescribed as empirical therapy. As alternative infectious causes were progressively excluded, treatment was narrowed. Contemporary European recommendations generally support substantially shorter treatment courses for Lyme neuroborreliosis [1,7]. Randomized data have demonstrated that oral doxycycline can be effective compared with intravenous ceftriaxone in European Lyme neuroborreliosis [10], and more recent data have not demonstrated additional benefit from prolonged antibiotic treatment [11].

The six-week ceftriaxone course administered in this patient therefore exceeded standard recommended treatment durations and should be regarded as an individualized multidisciplinary decision made in the setting of unusually severe and persistent CNS inflammatory abnormalities, rather than as a recommended standard regimen for Lyme neuroborreliosis.

The partial improvement in CSF inflammation and neuroimaging abnormalities during antimicrobial therapy provides support for an infectious inflammatory process but cannot establish *Borrelia* as the definitive cause. The absence of a *Borrelia*-specific antibody index remains the principal diagnostic limitation of this case.

Finally, the patient's death occurred following ventilator-associated pneumonia complicated by septic shock. This infectious complication developed in the context of persistent severe neurological deficits, prolonged impairment of consciousness, and extended invasive mechanical ventilation. Thus, although death was not directly attributable to Lyme neuroborreliosis, the prolonged neurological and ventilatory course likely contributed to the development of the ultimately fatal complication.

## Conclusions

Lyme neuroborreliosis should be considered in the differential diagnosis of unexplained inflammatory CNS disease, particularly when encephalopathy or focal neurological deficits are preceded by symptoms compatible with painful radiculitis. This case illustrates possible Lyme neuroborreliosis associated with an exceptionally unusual neuroimaging pattern characterized by intraventricular inflammatory material, leptomeningeal involvement, and focal parenchymal abnormalities suggestive of cerebritis. Routine CSF bacterial cultures, bacterial antigen testing, viral PCR assays, and *Borrelia* PCR were negative. However, a negative *Borrelia* PCR does not independently exclude Lyme neuroborreliosis.

This case also highlights the importance of distinguishing the detection of *Borrelia*-specific antibodies in CSF from definitive evidence of intrathecal antibody production. Because a paired CSF-serum *Borrelia*-specific antibody index was unavailable, intrathecal antibody synthesis could not be formally demonstrated, and the case fulfilled criteria for possible rather than definite Lyme neuroborreliosis. CSF serological findings should therefore be interpreted in conjunction with the clinical presentation, inflammatory CSF profile, neuroimaging findings, and exclusion of alternative diagnoses.
